# How Information Communication Technology Can Enhance Evidence-Based Decisions and Farm-to-Fork Animal Traceability for Livestock Farmers

**DOI:** 10.1155/2020/1279569

**Published:** 2020-12-17

**Authors:** G. Mwanga, E. Mbega, Z. Yonah, M. G. G. Chagunda

**Affiliations:** ^1^The Nelson Mandela African Institution of Science and Technology, Tengeru, Arusha, Tanzania; ^2^Department of Animal Breeding and Husbandry in the Tropics and Subtropics, University of Hohenheim, Garbenstr.17, 70599 Stuttgart, Germany

## Abstract

Due to changes in the livestock sector and the rise of consumer demand for comprehensive and integrated food security and safety, there has been a concern on the use of farm data in enhancing animal traceability and decision-making by farmers and other decision-makers in the livestock sector. To ensure high production through effective decision-making and auditable standards, producers are required to have better traceability and record systems. Therefore, this study aimed at (1) reviewing the current recording/data management and animal traceability systems used by small-scale farmers in developing countries and (2) analyzing how data management systems should be designed to enhance efficient decision-making and animal traceability from farm to fork. This study found that, still, a majority of small-scale farmers do not keep records leading to poor decision-making on the farm and policymaking. We also found that those who keep records do not store their data in electronic format, which again poses another challenge in data analysis. Moreover, this study found that the majority of traceability tools used by farmers in developing countries do not meet international standards based on tools they use for tracing animals; farmers were reported to use tools like branding and ear tagging, which provide very little information about the animal. Such tools lack the capability to keep track of useful information about an animal, e.g., information about feeding and animal health. In conclusion, this study recommended a better electronic system to be used at the farm level to facilitate data analysis, hence promoting informed decision-making and adherence to the international animal traceability standards. Otherwise, there is a need for researchers to conduct more studies in developing different analytical models for exploring on-farm data in order to improve the decision-making process by farmers and other stakeholders.

## 1. Introduction

Due to an increase in population and urbanization, it is estimated that in twenty years to come the demand for food including livestock products will double [1, 2]. This poses a question on how the livestock sector will meet the expected demand [3]. However, the expected increase in demands will open a window for a massive inter- and intra-trade and it also promises a major opportunity for poverty reduction and economic growth [4–6]. Moreover, the increase in income and urbanization will result in major structural changes in the entire livestock commodity chain, which has significant implications for the definition and control of food safety standards [7].

Currently, the production rate is not equivalent to the expected demand due to various challenges faced by the livestock sector including diseases, climate change, poor management systems (animal husbandry practices), genetics, farmer capacity and skills, marketing, infrastructure, and lack of information for proper decision-making [8–10]. In the context of this study, “decision-making” is the process through which alternative courses of action are sought, selected, and implemented to achieve business objectives [11]. It is advised that livestock sector should now consider developing and adopt better technologies and policies that will facilitate an increase in production [10, 11].

With regard to the previous argument, information was also identified as an important factor that will guide farmers in maintaining efficient use of farm resources [12]. Putting the right information in a timely manner within the hands of the farmers is considered as an empowerment mechanism for controlling their resources and managing a farm by guiding them in making evidence-based decisions [13–17]. In achieving all of the above, Capalbo et al. [18] discuss the critical need for data, models, and knowledge products (analytical tools) that will provide user-friendly information [18, 19] for decision-making. This needs to cut across from farm-level decision support to the agricultural research community and donors for making research investment decisions [20], to policy decision-makers [21] whose goal is to maintain a sustainable production [22].

On the other hand, global trade, diseases, traceability, intensification of production systems, and increased demand for safe animal products by consumers and food processors have increased the need for livestock identification and recording [23–25]. Moreover, traceability has become an important issue even to retailers who have also found that they can gain commercial advantage or maximizing sales by verifying their sources. Tracking of animal products has several advantages, i.e., reducing the time required to locate diseased animals and reducing exposure of healthy animals to the disease so minimizing as much as possible the impact of an outbreak of the disease on producers and international trade, e.g., how China had to impose a lockdown in Wuhan and other cities in an effort to quarantine the center of an outbreak of coronavirus disease 2019 (COVID-19) [26].

There is multiplicity of assurance schemes where producers who sell their raw products have to meet the standards [26, 27]. Also, it has been argued that nations/countries will not be able to meaningfully gain access to international markets without meeting minimum international disease control standards including animal traceability [28, 29]. Therefore, there is a need for the countries to put in place the necessary conditions to usher an appropriate animal identification system [30]. However, it has been reported that developing countries are being confronted with changes in the international rules governing trade in animal products [31]. Also it was argued that in order for countries and their stakeholders to maximize the benefits of globalization they must become familiar with, and must adhere to, the rights and obligations set out by the World Trade Organization (WTO) under the Agreement on Sanitary and Phytosanitary Measures (SPS) [31–33].

Animal identification involves identification, registration, and collecting data for each animal throughout its entire life cycle such that individual characteristics and the history of the animal can be traced back [24, 34]. Generally, the system should uniquely identify an animal and have a credible and verifiable mechanism for identity preservation [23]. Data that need to be collected include date and place of birth, ancestry, sex, geographic movement, health, and other production records for purposes of tracing the animal and its products [35]. Thus, a very intensive recording system is needed and necessary.

Nevertheless, it has been identified that the level of adoption and implementation of identification and recording in Africa is highly variable [25] and constrained by lack of infrastructure and facilities, insufficient expertise, and low awareness amongst stakeholders [35]. In implementing animal identification and recording systems, several questions have been raised, including what practical, simple, and cheap forms of identification and recording systems can be adopted? Is it Information Communication Technology infrastructure or use of traditional methods or can these traditional methods be incorporated into modern systems? To answer these questions, this paper reviews different studies that have addressed the issue of farm recording systems and animal identification.

## 2. Materials and Methods

The search for articles involved major two databases: ScienceDirect [36] and IEEE Xplore [37]. Also, other articles were searched in subject specific professional websites, newspapers databases, and Google Scholar which is a web scientific indexing service.

The first step involved searching for relevant articles. The study started by retrieving articles related to records keeping, data analysis, animal traceability, and identification on the farm including methods and tools used. Moreover, other articles related to livestock policy making and technology implementation were retrieved. In searching, the following keywords were used: “records keeping by farmers,” “records keeping methods,” “data analysis by farmers,” “data analysis tools,” “policy making,” “traceability and identification.” “Livestock” and “dairy” were used as a keyword for inclusion criterion. A relatively small number of studies exist from journals on the topics related to records keeping and farm data analysis; hence, others were searched from professional and newspapers sites. Moreover, the reference lists of each article were reviewed in detail to find additional articles.

## 3. Results and Discussion

### 3.1. On-Farm Data for Enhancing Decision Making

Table 1 summarizes all articles that were reviewed on the use of on-farm data. In this section, the status of farm records was reviewed, where information such as methods used by farmers to keep records and data reliability was retrieved. Also, various benefits that a farmer will get from keeping records were documented. However, we also reviewed how Information Communication Technology (ICT) can be used to assist farmers keeping and maintaining their farm data so they can add value to their farms.

#### 3.1.1. Importance of On-Farm Data

Records keeping at farm level have been considered as the most important management tool [13–17] that a farmer can use in improving their daily operations [14, 37, 38]. Keeping of records has immediate bottom line profitability of the livestock operation [39], where it assists a farmer in monitoring animal health, making rational decision, and proper monitoring of other farm management practices [40]. Moreover, it was identified that keeping of records has a relationship with farm production where farmers who keep records their farm has more production than those who do not keep record [39]. This can be due to the fact that farmers who keep records are more likely to use them for animal management and monitoring, planning of a farm, and improving farm management efficiency [41].

Also, it was highlighted that keeping of records has several advantages in performing genetic evaluation (farmers can use past records to select the best animal for breeding). Ghosh and Khan (2014) specified how access to on-farm information is significant in maximizing farm profit as follows:

1. Farmers can know exactly where they stand in relation to their optimum production goal by comparing input costs to benefits
2. Farmers can be able to identify early when problems with herd production and reproduction are rising so they can be corrected
3. Farmers can be able to quantify the effect of management changes they implement toward achieving their goals [41, 42]

#### 3.1.2. Status of On-Farm Record-Keeping

This study observed that in most developing countries the majority of farmers do not keep records [44, 45, 47]; regardless, it has been a practice of extension officers to emphasize farmers to keep records. While other farmers fail to produce any records, those who keep records have been using books, papers, and notebooks [47, 71] which presents a challenge in data analysis [48, 49]. Moreover, at some point folders or multiple books have been used especially for commercial farms [51], to separate different types of records; e.g., a farmer can use one folder to store all receipts of sales and another for expenses. This system might be perceived to be simple, but the disadvantage is that data is not well organized so when a farmer needs information often, he has to sort through piles of papers and do all computations manually. In addition, farmers have been claiming to keep records but in reality, they rely on their memory [46] and others used to write on the wall. Reasonably, these also do not provide a long-term solution, as to how these data can be integrated to provide information and it is very easy for a farmer to forget or for data to be damaged.

Moreover, the fact that farmers do not keep records has cost the African continent, especially developing countries, where it has been difficult even for policymakers to extract valuable information from farms [44]. Likewise, using unspecified animal recording books, and farmers behavior of not keeping records on a daily basis due to the nature of small-scale farming, has caused on-farm data being too fragmented to extract sufficient information [12].

Comparing small-scale and large-scale farming systems, most of the small-scale farmers do not keep records because a small-scale farmer is being engaged to many other activities [43], e.g., cropping, which also requires attention compared to large-scale farmers who dedicate much of their time and resources to one business. Other reasons that were reported are interest/do not see the importance [52], illiteracy, and low numeracy level especially in the lowest resources in African farming communities [12]. In such circumstances, most of small-scale farmers are challenged by poor records where data is fragmented or missing (incomplete) and due to other errors. Hence, most of them end up making uninformed decisions that affect their productivity [53].

However, it is being stated that a successful decision-making system goes beyond creating and displaying valuable data but integrating those records into operation (decision-making) [11, 72]. Data need to be processed into information and this requires analytics tools and sometimes even advanced techniques such as data mining and machine learning. Considering that a farmer makes decisions every day, the decision supporting system must be able to provide quick and efficient flow of information and knowledge to the livestock farmers [54]. Despite the role that analytical tools can play on the farm, converting data into information, very few studies have discussed this. However, the success of analytical tool depends on electronic data, which most of small-scale farmers fail to produce. The study that was done in India to compare the use of ICT framework shows that farmers who use ICT were making significantly better-quality decisions as compared to non-users [2]. Therefore, there is a need for integrating on-farm recording system to analytical tools, and hence it requires the power of ICT to enable a farmer to make evidence-based decisions.

#### 3.1.3. ICT as a Tool for Enhancing Decision-Making on the Farm

Different software for keeping on-farm records running on a computer has been proposed, e.g., Excel Spreadsheets, Quick Books, and AgSquared [53, 54]. However, complex programs such as Quick Books are not favorable to small-scale farmers as they are costly and a farmer will need intensive training. At some point, farmers will find them difficult to use and they will end up rejecting them. Excel Spreadsheets have been commonly used and many farming models have been implemented in Excel [56]. Nevertheless, a farmer needs to have a computer or a device that can run the program, the technology that is not accepted by many small-scale farmers as it is still expensive to own a computer and most farmers do not prefer as it is difficult to operate.

In integrating ICT in agriculture, many studies on small-scale farming systems have focused on market access [57], and little has been done on animal husbandry practices, e.g., records keeping. Initiatives that have been taken to link farmers into the market had shown significant improvement in market access. One of the media that has been used to link farmers directly to the market is mobile phones; others include radio and television. Likewise, the use of mobile or computer (referred to as M-Agriculture) is now recommended to be used in collecting data electronically [58].

Mobile devices such as cell phones, Personal Digital Assistants (PDAs), tablets, and other handheld communication tools are more recommended. An increase in the use of precision farming and mobile technologies along with improvements in data management software offers expanding opportunities for an integrated data infrastructure linking farm management decisions to site-specific bio-physical data. The reason why mobile technology is now preferred compared to Personal Computer (PC), especially in the developing world, is the cost of acquisition of a typical mobile phone is lower than that of a PC. It is also easy to learn how to use a mobile phone, even for computer-illiterate people. Also, it has been argued that the efficiency of production records-keeping and decision support is improved by a simple and friendly system [59]. This fact makes a mobile device the most appropriate medium to introduce technology to users who are not computer savvy. Another advantage of mobile phones is the high penetration of mobile phones in the developing world in the past decade. Compared to the number of PCs, mobile phones have a relatively higher infiltration level [60].

**Figure 1:**
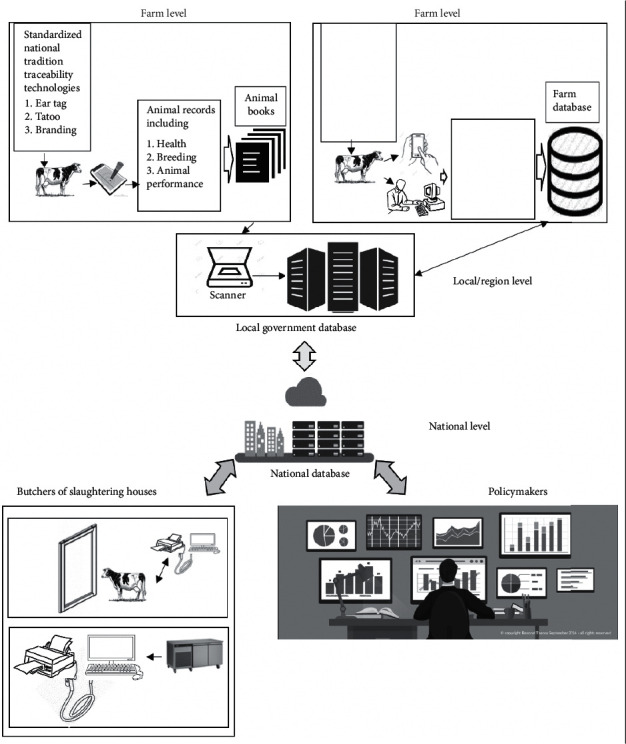
A proposed conceptual framework for a national traceability system and a data management system that will allow data collection from farm level (farmer) to national level (policymaker). The proposed framework comprises different tools and technologies, i.e., the use of mobile phones, traceability systems, and Internet that can be used for data collection and animal traceability and enhance evidence-based decision-making from farm to fork. The proposed framework will allow farmers to collected data electronical through the mobile devices or computer and this information will be stored to the local district/regional server, while other information from other stakeholders such as butchers or slathering houses will also be collected. All of this information will be aggregated to the national databases for policy and other decision-making.

However, using a mobile device as an interface to a farm recording system presents a number of challenges, one being poor network coverage in most of developing countries especially in the rural areas as most mobile operators concentrate on the densely populated urban areas before deploying good-quality network coverage in rural areas. Also, despite high mobile phone penetration in the developing and developed world, still limited memory capacity is a challenge, which is crucial in implementing recording system. Therefore, in consideration of implementing mobile application to support electronic recording system, these are the challenges that the livestock sector needs to improve, setting up good network infrastructures, local and national storage systems as presented in Figure 1.

Moreover, another recording technology that is widely used in developed countries especially in large-scale farms is RFID and sensors. These devices can be a target in one part of an animal which can be used to detect animal movements, nutrition requirements, and whether the animal is on heat or need to be vaccinated or deformed [60–64]. All information generated is sent to a farmer's mobile phone. Also, farmers have been using automated milking records systems to collect and analyze daily. This method has proven to be significant to farm production as a farmer can automatically monitor or identify the needs of his animals effectively.

#### 3.1.4. Data Analysis for Enhancing Decision-Making

The agricultural sector, like all parts of our global economy, is becoming data-rich due to advances in remote and mobile measurement technologies. By looking at what is being produced on dairy farms from sensors, Radio Frequency Identification Devices (RFID), and different activities/operations on the farm, a massive amount of data is produced daily [73]. This makes it even harder to manually retrieve information from collected data because there may be too much information to process. This implies that big data technology is soon going to be more common in dairy farms [74]. However, most of the study shows that the emphasis is on the data collection, and with that, a number of technologies for collecting data from the farm have been developed and many more to come. However, there is a gap in integrating these data and analysis coupled with decision-making tools that can assist decision-making, also for the fine-tuning of agricultural policies. Therefore, this poses a question to researches on how they can work with farmers in finding out different innovative technologies that can bridge this gap.

With regard to policymaking, big data is now unavoidable. Big data is believed to be a solution to many pressing economic and societal challenges. More and more companies and communities today realize that they are not going to be competitive if they cannot put their data to perform analytical, precision, logistics, decision-making, forecasting, and other tasks supported by Information Technology (IT) related services [75]. It is a growing trend in the IT sector serving different fields of knowledge including agriculture [66], in ensuring food safety, food sustainability, and crop improvement, marketing, and improving the food chain [67]. However, it is more realized in developed countries. In agriculture, big data helps to ensure digital farms, though for the digital farm to work it involves robust infrastructure including precision farming technologies, smart agriculture IoT (Internet of Things Technologies) solutions [68], wireless technologies, and cloud computing [69]. However, at some point it requires higher-speed connections [70], consistent data linkage, good security, and capacity to process large amounts of data in real time between machines and the cloud.

By using farm data to drive input management and other farm decisions, policymakers, other livestock stakeholders, and producers can be able to identify and quantify limiting productivity variables. However, using these data for policymaking requires good analytical or data mining technologies. Machine-learning systems infer patterns, relationships, and rules directly from large volumes of data in ways that can far exceed human cognitive capacities. Despite the fact that machine learning has been around for a number of decades and considered as a new engine for economic growth, still most organizations have not truly grasped how machine learning will change the way they do business, and this includes livestock industry [75].

### 3.2. Animal Traceability

#### 3.2.1. What Is Animal Traceability?

Table 2 summarizes all the articles that were reviewed in regard to animal traceability. Animal traceability and identification can be defined as linking of components including identification of establishments/owners, the person(s) responsible for the animal(s), animal movements, and other animal records, i.e., feeding, breeding, and health records. Moreover, it can mean identifying a group of animals with a unique group symbol or identifier, i.e., animals belonging to residents of a village, or animals sharing a communal animal handling facility. Thomson et al. [76] stated that “International trade, disease control, and consumer confidence depends on the accountability and traceability that an animal identification system could provide.”

Animal traceability and identification have been practiced for over 3800 years [98], where farmers use body marking as a means of animal traceability. Also, they used a red-hot iron to brand their animals, and this was principally used on valuable animals, in particular horses. Branding for disease control purposes commenced later. However, modern traceability was not available; hence, farmers use indelible branding and strict health certification. Also, during disease outbreaks, animal products were closely monitored and some animal products could not be traded internationally unless accompanied by a certificate of origin guaranteeing safety [98].

Animal traceability brings up the ability to follow an animal or group of animals during all stages of their life [74, 99], i.e., from the food chain, from producer to slaughter or to retail and tracing, which in other terms is referred to as the ability to follow a meat product up to the supply chain by means of the records which have been kept at each stage of the chain. According to International Organization for Standardization (ISO), traceability is defined as the ability to trace the history, application, or location of what is under consideration or a series of recorded identifications. Experience has shown that a traceability system can reduce the time required to locate diseased animals and reduce exposure of healthy animals to the disease so minimizing as much as possible the impact of an outbreak of the disease on producers and international trade.

A successful traceability system is essentially relying on record-keeping for success. However, this differs among countries where others like UK, in Northern Ireland, have established a system which is fully integrated with the Animal and Public Health Information System [100]. It can record movements and other data for all farmed livestock. In places where they have failed to integrate the traceability and recording system, their data quality is insufficient to allow full exploitation of a potentially valuable resource [78]. Therefore, there is a need for integrating the two systems.

The minimum required information that a farmer needs to record includes animal's place and date of birth, the name and address of the owner, the date and location of movements between the animal's origin and its place of slaughter, and the date and location of slaughter [79]. The more elaborative system ID system should contain animal's place and date of birth, the name and address of the owner, animal feeding, animal watering, veterinary drugs, farm management, preparation of animal for slaughter, common measures for records keeping and traceability [80], the date and location of movements between the animal's origin and its place of slaughter, and the date and location of slaughter [81].

#### 3.2.2. Animal Traceability and Recording System Framework: From Farm to Fork

Figure 1 shows the proposed conceptual framework for a national data management system for enhancing nation and international animal traceability and policymaking. Based on the context of animal traceability, that is a tool to help countries meet their objectives of controlling, tracing an animal from farm to fork, and preventing and eradicating animal diseases [25, 77, 98]. In choosing a system, it is recommended that various factors need to be considered because it is noted that there is a wide variability amongst systems worldwide and these are attributed to the differences in sanitary and economic or sociocultural criteria and also the level of development and implementation of animal welfare differs from one country to another [82]. Therefore, it is recommended that the process of developing this system should begin on international harmonization [83].

The second thing to consider while setting up a traceability system is to identify different characteristics that need to be traced throughout the various steps in the food production chain [84]. Quality assurance programmers aim for a “whole of chain” approach, so that the system is implemented “from farm to plate,” i.e., from on-farm practices to the refrigeration, storage, and transportation stages [85]. It is recommended that until the animal has been taken to the slaughtering house, primary production records should be able to be retrieved. This information makes it possible to carry out risk-based inspections [86]. However, for the tradition animal traceability, they can use the slaughtering house veterinarian as a professional devoted to providing care to animals, to ensure the good standard of an animal [86]. The method does not really guarantee the quality.

The components of a traceability system should include the following: biosecurity, disease monitoring, and reporting, feedstuff safety, the safe use of agricultural and veterinary chemicals, the control of potential food-borne pathogens, and traceability [87, 88]. These can be controlled from the farm by keeping records including animal health records, breeding, and animal feeds [89].

It is recommended that there is a need of integrating the farm recording system to the international traceability systems during the second stage when an animal is sold to the retailers [74, 98].

However, there has been a question of how it should be implemented and who is involved. The USDA has allowed each state/tribe to implement its own traceability system, by setting basic guidelines where each state/tribe runs its own database [101]. They have allowed a wide range of products to be considered official devices ranging from a simple metal tag with official numbers to RFID tags that are integrated into other production systems [90]. But then again, Bénet et al. [91] argued that the Veterinary Authority, with relevant governmental agencies and in consultation with the private sector, should establish a legal framework for the implementation and enforcement of animal identification and animal traceability in the country.

Also, others argued that establishing animal traceability system involves a number of different stakeholders and in that case veterinary has also been considered as a central responsibility in international trade in animals and animal products [30, 86]. However, the whole process has to start from the farm; a farmer is responsible for keeping records. Moreover, the established legal framework should include elements such as the objectives, scope, organizational arrangements including the choice of technologies used for identification and registration, obligations of all the parties involved, third parties implementing traceability systems, confidentiality, accessibility issues, and the efficient exchange of information [91].

However, the technology keeps on advancing and instead of farmers using their traditions methods they adopted computerized systems such as the use of computer databases to keep farm records [92]. Chips and sensors that read animal information enhance the speed and accuracy of data acquisition and manipulation [23]. Also, the accelerating pace of innovation and development within the field of molecular genetics have invested a more advanced technology of using DNA testing technologies to issues of traceability of live animals and derived products [93].

#### 3.2.3. Mechanical System for Animal Traceability and Recording

There are various animal identification systems. Ekuam [30] classified these systems into three groups including mechanical, biological and electronic. Mechanical system includes paint marks, ear tags (plastic), ear tags (metal), ear notching, drawings, and descriptions and photographs. Paint mark is mostly used in the auctioning markets; mostly, it cannot be used in the farm due to the reason that it is not permanent (it can be removed easily). However, when you compare ear tags and other methods, ear tags have been the mostly used method, especially in the private farms because they are cost-effective, and they are more visible from afar. Metal tags are considered to be more durable than plastic ear tags and do not tear out of the ear easily. However, they are mostly used by small stock farmers and they are difficult to read from a distance.

Ear notching is a numbering system that ranges from 1 to 1,690 where the animal is uniquely identified by a combination of numbers depending on the number and position of notches cut in the ear. Drawing as a method of identification is identified at an early stage by drawing the unique color patterns onto a pre-printed sheet issued by the breeders' “society.” Another method related to drawing is animal branding; this method is widely adopted by pastoralists, e.g., Maasai [94]. It involves the burning of an identifying mark into the hide of an animal as letters, pictographs, and symbols. Their combination using freeze or hot iron branding has been the only method of marking on the animal that lasted for the life of the animal until the invention of the tattoo [30]. But, this method is limited to breeds with distinctive coloration and/or color patterns. Generally, all mechanical identification systems are not secured; they are very easy to tamper with and mostly they provide little value as evidence in the eyes of the law. More details on the mechanical identification system can be found in the study done by Ekuam [30].

Electronic systems include microchips and RFID. A microchip implant is an identifying integrated circuit placed under the skin of an animal. It is a passive RFID device. Lacking an internal power source, it remains inert until it is powered by the scanner. It has become a law to monitor dogs in UK that, due to the number of lost and stray dogs, from 6th April 2016 it is now compulsory for all dogs in the UK to be microchipped and registered on a government compliant database [95]. This identifies an animal electronically where information about an animal is stored electronically. However, this method poses a number of problems; that is, microchips tend to migrate in the body and sometimes cause abscesses as the implantation is done with a special tool penetrating the skin and this poses a major threat to the meat industry.

The United States Department of Agriculture (USDA) has finalized the rules for improving the traceability of US livestock moving interstate [90]. Among the things that were agreed on is that each US state or tribe implements the law independently within the framework established by the federal government where it allows a wide range of products to be considered official devices ranging from a simple metal tag with official numbers to Radio Frequency Identification Device (RFID) tags that are integrated into other production systems [90].

Tagging livestock with RFID can be an important tool in a farmer's arsenal to identify each animal along with its pedigree and medical information. Using RFID, a veterinary officer can just scan the animal during veterinary visits or inventory counts, and with the help of software, it uploads significant information from each animal to a database. In addition to that, there are newly invented RIFD devices called Low RFID which use UHF RFID and GPS to track the animal's movement in order to identify feeding and travel habits, and even monitor heart rates [97]. This method has been considered as the potential animal identification system; however, the major problem with using this technology as a national identification method is the cost [96].

Moreover, for bio-identification, it was identified that photographs do not qualify as a bio-identification method because they are subject to changes as time goes by. Therefore, biological methods including nose prints, iris prints, and DNA analysis can be used to uniquely identify an animal. Nose prints involve the making of a scanable print of the animal's nose shield and the image is stored on a computer for future reference. Likewise, although iris prints are still in their infancy, it has been found that they will probably be used to only identify animals with a high monetary value. Moreover, DNA analysis involves a laboratory analysis of 12 micro-satellites from the unique DNA found in the nuclei of cells in a biological sample such as hair, blood, or meat. This method has been proven by the courts as a scientifically proven method of identification [30].

There are a number of economic and infrastructural challenges regarding the applicability of traceability in developing countries [30, 32], especially in the pastoral livestock systems. The cost of applying a standardized and harmonized identification and traceability system is unaffordable. This is compounded by the disparity of agricultural production systems. The low volume of production output by many producers/farmers/livestock keepers implies that harmonization is difficult to achieve. However, it was once advised that developing countries have the option of forming working groups with partner states affected by the same problem in order to take advantage of economies of scale in livestock identification and traceability to enhance their export market potentials, increase production and productivity of the pastoral areas, and develop unified infrastructure to achieve equality in compliance with respect to livestock identification and traceability.

## 4. Conclusion

Data quality and sufficiency are the preconditions for making rational decisions which in turn reflect on the farm production. However, most of small-scale farmers are challenged with poor records. While other farmers fail to produce any records, those who keep them have been using poor recording system and mostly their data is fragmented or missing (incomplete) which presents another challenge in data analysis. Based on the results from this study, it is obvious that keeping of records is no longer an option for a farmer. It has also been identified that due to poor recording systems it has cost the African continent, especially developing countries, where it has been difficult even for policymakers to extract valuable information from farms. Therefore, there is a need of educating farmers on the essence of keeping records. Moreover, it is also recommended that the government can start initiatives for mobilizing livestock data from the farm on a daily basis. This goes along with setting of ICT infrastructures that can link a farm to the national database.

Moreover, increased demand for safe animal products by consumers and food processors has increased the need for livestock identification and recording. International trade, disease control, and consumer confidence depend on the accountability and traceability that an animal identification system could provide. Based on the fact that animal traceability requires following of an animal or group of animals during all stages of their life to the supply chain by means of the records which have been kept at each stage of the chain, it is obvious that a successful traceability system is essentially relying on record-keeping.

This study has demonstrated that one of the proposed solutions is the adoption of electronic devices for collecting information, traceability, and other on-farm data. It is recommended that, instead of farmers using their traditional recoding system, i.e., papers and relying on their memory, they should adopt electronics systems for easy storage and accuracy of data acquisition and manipulation. However, countries, especially developing countries, should also think of implementing an integrated animal records and traceability system at the national and international level. This will enhance evidence-based decisions and policymakers will be able to establish policies based on what is being observed from the farm. Moreover, if farmers can be in a better position by adopting electronics tools for traceability such as RFID or animals' sensors, they are advised to do so, as these will assist the farmers in keeping track of their animals in a more efficiency way. This information can even be shared with the market and add more value to their animals but also as one way for them to comply with the international market standards. In addition, there is a need of increasing awareness, advocacy, understanding, and technical capacity in Africa to address the constraints related to animal identification and recording, which are now negatively impacting the competitiveness of animal products from Africa.

## Figures and Tables

**Table 1:** Reviewed articles on-farm data: importance of on-farm data, status of on-farm records and the use of ICT for enhancing decision-making.

Topic	Number of articles	Authors
Importance of on-farm data		Maningas et al. [13]; Birkhaeuser et al. [15]; Cash [16]; Galloway and Mochrie [17]; Adhiguru et al. [14]; Figurek [38]; Hunter [36]; Yeamkong et al. [39]; Silver [40]; Rhone et al. [41]; Ghosh and Khan [42]; Minae et al. [43]; Ali [44]
Status of on-farm record-keeping		Dudafa [45]; Chagunda et al. [46]; Acuña and Petrantonio [47]; Dudafa [45]; Brooks-Pollock [48]; Kwame, Appiah et al. [49]; Thornton [50]; Adrian [51]; Gichohi [12]; Minae et al. [43]; Tebug et al. [52]; Kwame et al. [53]; CIMA [11]; Grisham and Gillespie [54]; Pica-Ciamarra et al. [2]
ICT as a tool for enhancing decision-making on the farm		Grisham and Gillespie [54]; DAIRY-CATTLE [55]; Gibbs et al. [56]; Birthal et al. [57]; Tembo and Maumbe [58]; Lia et al. [59]; Gichamba and Lukandu [60]; Farmnote [61]; Huddleston [62]; Anderson et al. [63]; Connecterra [64]; Moocall [65]
Data analysis for enhancing decision-making		AIMS [66]; Talascend-LLC [67]; PTC [68]; Satej and Suresh [69]; Brian [70]

**Table 2:** Reviewed articles on animal traceability: animal traceability (from farm to fork) and recording system framework and mechanical system for animal traceability and recording.

Topic	Number of articles	Author(s)
What is animal traceability?		Thomson et al. [76]; MLD [35]; Morgan et al. [77]; Small [78]; Scottish [79]; Oie and FAO [80]; Greene [81]
Animal traceability and recording system framework: from farm to fork		Barcos and Pettitt [27]; Pettitt [27]; Rojas et al. [82]; Barcos [83]; Ammendrup and Barcos [84]; Butler et al. [85]; Schnöller [86]; Dagg et al. [87]; Thomson et al. [88]; Romero et al. [89]; Greene [81]; Small [78]; Ahmed et al. [90]; Bénet et al. [91]; Le Brun [31]; Houston [92]; McKean [23]; Cunningham and Meghen [93]
Mechanical system for animal traceability and recording		Ekuam [30]; Nanzala [94]; Ekuam [30]; TRACER [95]; Ahmed et al. [90]; Tonsor and Schroeder [96]; Insider [97]; Ekuam [30]; Le Brun [31]; Perry and Grace [33]
